# *Helicobacter pylori* infection affects the human gastric microbiome, as revealed by metagenomic sequencing

**DOI:** 10.1002/2211-5463.13390

**Published:** 2022-03-25

**Authors:** Daoming Wang, Tongda Zhang, Yueqi Lu, Changzheng Wang, Yumei Wu, Jiandong Li, Ye Tao, Le Deng, Xiaoyin Zhang, Jinmin Ma

**Affiliations:** ^1^ BGI‐Shenzhen Shenzhen China; ^2^ Department of Gastroenterology National Clinical Research Center of Infectious Disease The Third People’s Hospital of Shenzhen The Second Affiliated Hospital of Southern University of Science and Technology Shenzhen China

**Keywords:** gastric diseases, gastric microbiome, gastric ulcer, gastritis, *Helicobacter pylori* infection, metagenome

## Abstract

*Helicobacter pylori* infection is a prevalent infectious disease, associated with many gastric diseases, including gastritis, gastric ulcer, and gastric cancer. To reveal the characteristics of the gastric microbiome in patients infected with *H. pylori*, we performed metagenomic shotgun sequencing of stomach swab samples from 96 patients and then conducted metagenomic association analyses between alterations in the gastric microbiome and *H. pylori* infection status. The overall composition of the gastric microbiota in *H. pylori‐infected* individuals was distinctly different from the negative controls; *H. pylori* became the dominant species after colonizing the human stomach and significantly decreased the α‐diversity of the gastric community (*P* < 0.05, Wilcoxon rank‐sum test). We also identified 6 HPI‐associated microbial species (FDR < 0.05, Wilcoxon rank‐sum test): *Stenotrophomonas maltophilia*, *Stenotrophomonas* unclassified, *Chryseobacterium* unclassified, *Pedobacter* unclassified, *Variovorax* unclassified, and *Pseudomonas stutzeri*. Furthermore, 55 gastric microbial pathways were enriched in the *H. pylori*‐positive group, whereas only 2 pathways were more abundant in the *H. pylori*‐negative group: dTDP‐L‐rhamnose biosynthesis and tetrapyrrole biosynthesis (FDR < 0.05, Wilcoxon rank‐sum test). Gastritis was not associated with non‐*H. pylori* species in the stomach (*P* > 0.05, Wilcoxon rank‐sum test). This study revealed alterations in gastric microbial taxa and function associated with HPI in the Chinese population, which provides an insight into gastric microbial interactions and their potential role in the pathological process of gastric diseases.

## Graphical abstract

Wang et al. performed shotgun metagenomic sequencing on gastric endoscopy samples from Chinese patients with gastric symptoms and then performed metagenomic analysis. The data suggest that *Helicobacter pylori* infection is associated with gastric microbial taxonomic and functional alterations, providing insights into gastric microbial interactions and their potential role in the pathological process of gastric diseases.

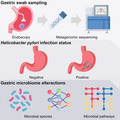

**Abbreviations**
CNGBdb: China National GeneBank DataBase
FDR: false discovery rate
HPI: *Helicobacter pylori* infection
PCoA: principal coordinate analysis
PERMANOVA: permutational multivariate analysis of variance
SD: standard deviation

*Helicobacter pylori* is the first bacterium that was found to be able to colonize and survive in the highly acidic environment of the stomach, and it is also the first confirmed prokaryote carcinogen [1]. *H. pylori* is a microaerobic Gram‐negative bacterium, which was isolated for the first time from patients with chronic active gastritis in 1984 [2]. *H. pylori* can infect the human host from childhood through an unsanitary diet or family transmission [3, 4], and persistently colonize the host gastric mucosa. The infection of *H. pylori* can lead to many gastrointestinal diseases, including peptic ulcer disease, atrophic gastritis, and gastric carcinoma [5], and is also associated with many extragastric diseases such as iron deficiency anemia [6], vitamin B12 deficiency [7], osteoporosis [8], and cardiovascular diseases [9]. As one of the most prevalent pathogens, *H. pylori* has infected nearly half of the global population [10], and the prevalence of *H. pylori* infection correlates with socioeconomic factors [11].

For a long time before the discovery of *H. pylori*, the highly acidic stomach was thought to be free of microorganisms, but the subsequent discovery of *H. pylori* changed this perception and further studies revealed that the human stomach also has a unique microbial community. The dominant gastric bacteria at the phylum level are *Proteobacteria*, *Firmicutes*, *Actinobacteria*, and *Bacteroidetes* [12, 13]. *H. pylori* colonization significantly alters the overall microbiome structure of the stomach, which in turn has an impact on host health. The bacterial number of *Proteobacteria* and *Acidobacteria* was higher in the stomach of *H. pylori‐infected* patients than that of *H. pylori*‐negative individuals [14]. The colonization of *H. pylori* decreased the microbial species diversity in the stomach, which may be caused by the preference of *H. pylori* for stomach pH. Several previous studies had preliminarily revealed that HPI may correlate with gastrointestinal microbiota shifts, and the *H. pylori* infection‐related alterations in the gut microbiome were found to correlate with the risk of out‐gastric diseases, for instance, vitamin B12 deficiency [15] and gastric lesion [16].

Previous *H. pylori* infection‐related gastric microbiome studies that used 16S amplicon sequencing may be limited by inadequate resolution for microbial taxonomy, and the primer selection and PCR amplification can introduce bias in the quantification of taxa abundance [17, 18]. Therefore, whether there are more bacterial species associated with *H. pylori* infection, and how does the gastric microbiome shifts associated with gastric diseases remain unclear. In addition, although the important role of the gut microbiome in human health on diseases has been comprehensively revealed, how the gastric microbiota interacts with the host and each other still lacks investigation at the microbial functional level.

In this study, we performed shotgun metagenomic sequencing in 96 patients from the Chinese population, aiming to compare the gastric microbial community structure between *H. pylori‐positive* and *H. pylori*‐negative individuals and identify the gastric microbial species associated with HPI. Association analyses with gastric diseases and the alterations in gastric bacterial functions were conducted to explore the potential physiological effects of *H. pylori* infection mediated by gut microbes, which may provide clues of how *H. pylori* influence the host health status.

## Methods

### Participants and samples

The patients with *H. pylori* examination were recruited in the Third People’s Hospital of Shenzhen, Shenzhen, China. The patients accepted gastric endoscopy examinations because of upper abdominal pain, urea breath test‐positive or other gastric symptoms. In this study, gastric swab samples were collected by endoscopy from the gastric locations with lesions, for instance, erosion, ulcer, or hyperplasia. The age and gender information of participants involved in this study is available in Table S1.

### Ethical approval

Written informed consent was obtained from the patients in accordance with the Declaration of Helsinki, and this work has been approved by the Institutional Review Board of BGI (BGI‐IRB 20170‐T1), Shenzhen, China.

### Sample pretreatment and DNA extraction

Genomic DNA was extracted from the gastric swab using the MagPure Tissue & Blood DNA KF Kit (MAGEN, Guangzhou, CHN). Genomic DNA concentration was determined by Qubit fluorometer (Invitrogen, Carlsbad, USA) and Qubit dsDNA HS Assay Kit (Invitrogen, Carlsbad, USA).

### Metagenomic sequencing and quality control

We constructed barcoded, paired‐end libraries with an insert size of ~ 250 bp for each sample using the MGIEasy Universal DNA Library Prep Kit (MGI, Shenzhen, CHN). About 500 ng genomic DNA of each sample was used to prepare sequencing libraries. The libraries were then multiplexed, and paired‐end (100 bp) sequencing was performed on the DNBSEQ‐T1 platform (MGI, Shenzhen, CHN).

We removed the human genome‐contaminated reads and low‐quality reads from the raw metagenomic sequencing data using kneaddata (version 0.7.4), bowtie2 (version 2.3.4.3) [19], and trimmomatic (version 0.39) [20]. In brief, the data cleaning procedure includes two main steps: (a) discard the human genome‐contaminated reads by aligning raw reads to the human reference genome (hg38) and (b) remove adaptor sequences using Trimmomatic. The cleaned metagenomic sequencing data are publicly available from the China National GeneBank DataBase (CNGBdb, https://db.cngb.org) via accession number CNP0002673.

### Taxonomic analysis

We generated the taxonomic relative abundance from the cleaned metagenomic reads using MetaPhlAn2 with default parameters [21]. MetaPhlAn2 uses unique clade‐specific markers to detect the taxonomic clades present in metagenomic sequencing data and estimate the relative abundance of the clades.

Alpha diversity and richness were estimated by the Shannon index and species number using the R package vegan. Differences in alpha diversity and richness were assessed using the Wilcoxon rank‐sum test. Beta diversity between groups was assessed using the Bray–Curtis distance and further visualized using principal coordinate analysis (PCoA) plots. The differential abundance of taxa between groups was identified using the Wilcoxon rank‐sum test. Before the comparison analysis, the low prevalence species (prevalence rate < 20%) were removed from the taxonomic relative abundance profile, and the relative abundance was recalculated based on the remaining species. The false discovery rate (Benjamini–Hochberg FDR, q < 0.05) method was used to adjust the *P*‐values for multiple test corrections. We conducted a pairwise correlation analysis between all gastric species using Spearman’s correlation and visualized the coabundance network using cytoscape (version 3.8.0).

### Functional analysis

We generated the relative abundance profiles of microbial metabolic pathways of all samples using the HUMAnN2 pipeline with default parameters [22]. HUMAnN2 maps cleaned metagenomic reads to the UniRef90, MetaCyc, and MinPath databases together with MetaPhlAn2 and ChocoPhlAn pangenome databases to quantitate the coverage of microbial gene families and pathways in metagenomic samples.

We calculated the Bray–Curtis distance of microbial pathway relative abundance profile between all samples using the *vegdist (method* = *‘bray’)* function from R package vegan (version 2.5‐6). Then, we performed PCoA based on the between‐sample distance matrix using the *cmdscale* (*k* = *5, eig* = *T*) function from the R package vegan. For the between‐group comparison analysis, we removed the low prevalent pathways (prevalence rate < 20%) and recalculated the relative abundance of remaining pathways by summing up to 1; then, we compared the differences in the relative abundance of microbial pathways between *H. pylori‐*positive and *H. pylori*‐negative groups using the Wilcoxon rank‐sum test. To control the false discovery rate (FDR), the Benjamini–Hochberg *P*‐value corrections were performed using the *p.adjust()* function in R.

## Results

### Overview of metagenomic sequencing reads

To investigate the characteristics of the gastric microbiome, stomach swab samples of 96 patients were collected using the gastric endoscope. Gastric endoscope examination diagnosed 66 patients with gastritis, of which 12 with chronic active gastritis and 54 with chronic gastritis. The mean age of the 96 patients was 47.98 years (22‐72, SD = 11.91), and the male/female ratio was 1.04. Metagenomic shotgun sequencing was performed on the 96 stomach swab samples using the BGISEQ‐500 platform and generated 13.47 million paired‐end reads per sample on average. After trimming and removing host‐derived reads, on average 8.78 million clean reads per sample were obtained for downstream analysis. On average, reads from the human genome accounted for 34.82% of total reads, which were caused by the high proportion of human tissue in stomach swab samples.

### *H. pylori* infection decreased the diversity of the gastric microbiome

**Fig. 1:**
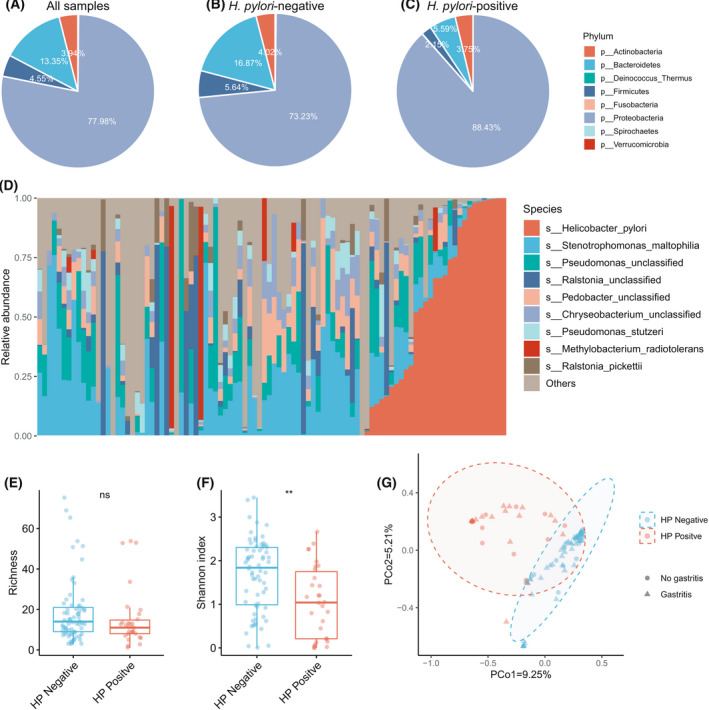
Gastric microbial composition and ecological indices in *H. pylori*‐positive and *H. pylori*‐negative individuals. (A)‐(C). The phylum‐level average composition of the gastric microbiome in all (A), *H. pylori*‐negative (B), and *H. pylori‐positive* samples (C). (D) The species‐level composition of the gastric microbiome in all samples, which were sorted by the relative abundance of *H. pylori*. (E) The species‐level richness of gastric microbiome in *H. pylori*‐positive and *H. pylori*‐negative groups. (F). The species‐level Shannon index of the gastric microbiome in *H. pylori*‐positive and *H. pylori*‐negative groups. (G) Principal coordinate analysis plot of species‐level gastric microbiome composition based on the Bray–Curtis distance. ns: insignificant (Wilcoxon rank‐sum test, *P* > 0.05); **: significant (Wilcoxon rank‐sum test, *P* < 0.01).

We generated the taxonomic relative abundance profile using metaphlan2 and found gastric microbiome was dominated by *Proteobacteria* (77.98%), *Bacteroidetes* (13.35%), *Actinobacteria* (3.94%), and *Firmicutes* (4.55%) (Fig. 1A); all these 4 dominant phyla accounted for 99.82% of the total reads. In the *H. pylori‐negative* group, the relative abundance of phyla *Bacteroidetes*, *Firmicutes*, and *Actinobacteria* was higher than in the *H. pylori‐negative* group (Fig. 1B,C, Wilcoxon rank‐sum test, FDR < 0.05), while the relative abundance of *Proteobacteria* was higher in *H*. the *pylori‐positive* group.

At the species level, we firstly investigated the relative abundance of the top 9 most abundant species and sorted the samples by the relative abundance of *H. pylori* (Fig. 1D). We found that the dominant species is different in the gastric microbial communities of *H. pylori*‐positive and *H. pylori*‐negative individuals. The relative abundance of *H. pylori* in the stomach dramatically increased in *H. pylori‐*infected individuals and dominated the gastric microbiome (Fig. 1D), While *Stenotrophomonas maltophilia* and *Pseudomonas* unclassified dominate the gastric microbial community in *H. pylori‐negative* individuals.

To investigate the effect of *H. pylori* infection on overall gastric microecological structure, we compared the ecological indices of the gastric microbiome between *H. pylori‐positive* and *H. pylori*‐negative groups, including richness evaluated by observed species number and alpha diversity evaluated by the Shannon index. The species richness showed no significant difference between the *H. pylori‐negative* and *H. pylori*‐positive groups (Wilcoxon rank‐sum test, *P* > 0.05; Fig. 1E). However, the observed alpha diversity in the species level was significantly higher in the *H. pylori‐negative* group (Wilcoxon rank‐sum test, *P* < 0.05; Fig. 1F).

To estimate the overall characteristic differences in gastric microbial communities between groups with beta diversity, we evaluated the between‐sample dissimilarity using the Bray–Curtis distance, which was further visualized in principal coordinate analysis (PCoA) plots (Fig. 1G). The total diversity captured by the top two principal coordinates was 5.72 and 1.88% for the Bray–Curtis distance. We estimated the explained variance proportion of gastric microbiome composition by age, gender, gastritis, and *H. pylori* infection status using permutational multivariate analysis of variance (PERMANOVA) and found that only age and *H. pylori* infection significantly influence the gastric microbiome composition (PERMANOVA, *P* < 0.05) and the gastric microbiome composition of *H. pylori*‐positive individuals was significantly different from negative individuals (PERMANOVA, *P* < 0.001). However, gastritis was not associated with the overall composition of the gastric microbiome.

### *H. pylori* infection altered the microbial interaction network within the stomach

**Fig. 2:**
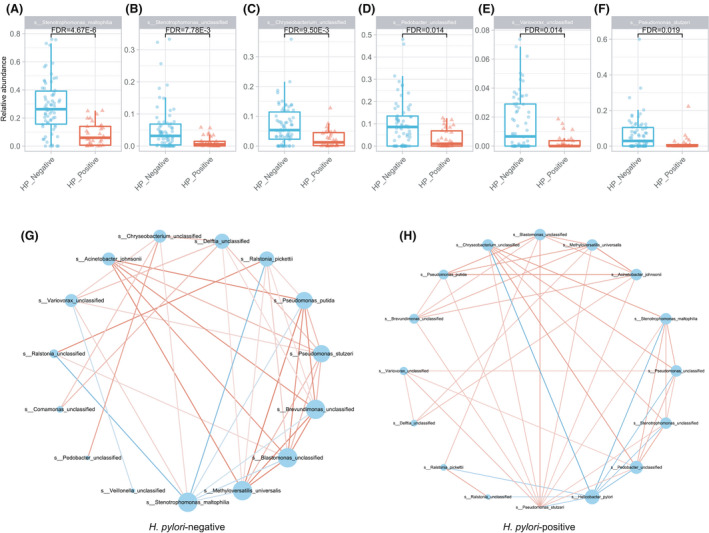
Perturbated gastric microbial community in *H. pylori‐infected* individuals. (A)‐(F). Gastric species with significantly different relative abundance between *H. pylori‐positive* and *H. pylori*‐negative groups (Wilcoxon rank‐sum test). (G‐H). Between‐species coabundance network in *H. pylori‐negative* (G) and *H. pylori*‐positive groups (H). The size of the dots in the network represents the number of edges.

Next, we investigated the effect of *H. pylori* infection on the relative abundance of non‐*H. pylori* gastric species, and how the *H. pylori* colonization affects the between‐species interaction in the gastric microbial community. We first revealed the non‐*H. pylori* species that displayed significant relative abundance between *H. pylori*‐negative and *H. pylori*‐positive groups (Wilcoxon rank‐sum test, Benjamin–Hochberg‐corrected *P* < 0.05; Fig. 2A‐F; Table S2); *Stenotrophomonas maltophilia*, *Stenotrophomonas unclassified*, *Variovorax unclassified*, *Chryseobacterium* unclassified, *Comamonas* unclassified, and *Pseudomonas stutzeri* were significantly enriched in the *H. pylori*‐negative group (Wilcoxon rank‐sum test, Benjamin–Hochberg‐corrected FDR < 0.05; Fig. 2A‐F; Table S2).

We then constructed the between‐species coabundance network in *H. pylori‐negative* and positive groups separately using the Spearman correlation. In the *H. pylori‐negative* group, most species were positively associated with other species, and most of the negative relationships were dominated by *Stenotrophomonas maltophilia* (Fig. 2G). In the *H. pylori‐positive* group, the negative relationships of *Stenotrophomonas maltophilia* with other non‐*H. pylori* species turned positive, and all the negative relationships were with *H. pylori*; the gastric communities were restructured by *H. pylori*.

### No correlation was observed between gastritis and gastric microbiome

**Fig. 3:**
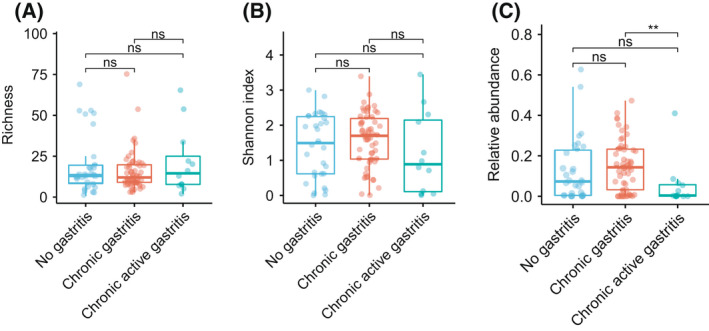
Alpha diversity and species abundance correlation to clinical parameters. There is no correlation between gastric microbiome species alpha diversity and gastritis (A‐B). Only the relative abundance of *Bacteroidetes* is negatively correlated with chronic active gastritis (C). The Kruskal–Wallis test was used, ‘ns’ means ‘insignificant’; ** means *P* < 0.01.

*H. pylori* infection is one of the leading causes of gastritis; however, whether other gastric microbial species also correlate with the onset and development of gastritis remains unclear. Thus, we compared the overall microbial ecological indices in stomachs between individuals with gastritis and those without gastritis. The patients with gastritis were divided into chronic gastritis and active chronic gastritis groups according to the gastric endoscope examination diagnosis. However, no significant difference in richness and the Shannon index of the species‐level gastric microbiome was observed between no gastritis, chronic gastritis, and active chronic gastritis groups (Wilcoxon rank‐sum test, *P* > 0.05; Fig. 3A,B; Table S3). We also compared the taxonomic relative abundance between 3 groups in the phylum level; only *Bacteroidetes* showed a significant difference in relative abundance between chronic gastritis and chronic active gastritis groups (Kruskal–Wallis test, *P* < 0.05; Fig. 3C).

### Functional alterations in the gastric microbiome were associated with *H. pylori* infection

**Fig. 4:**
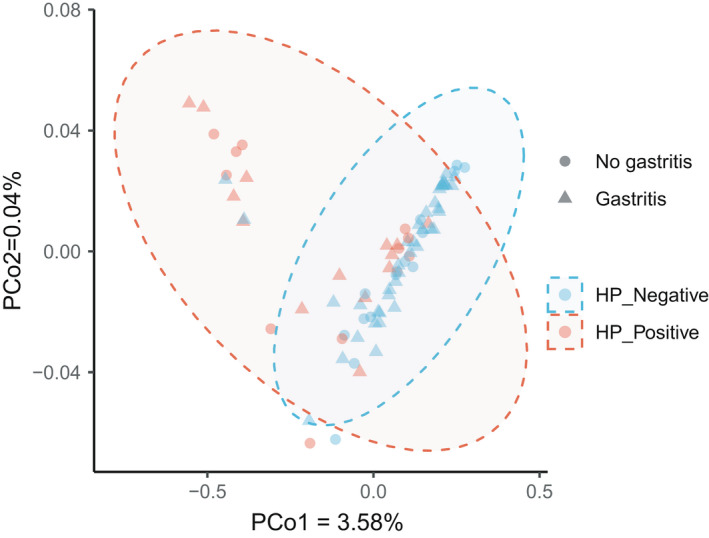
Principal coordinate analysis plot of gastric microbial pathways based on the Bray–Curtis distance.

In total, we identified 417 microbial metabolic pathways in all gastric metagenomic samples using HUMAnN2, then filtered out the pathways with a low prevalence (< 20%), leading to 237 microbial pathways for downstream analysis (Fig. 4). We compared the relative abundance of microbial pathways between *H. pylori‐positive* and *H. pylori*‐negative groups using the Wilcoxon rank‐sum test and identified 54 microbial metabolic pathways that showed a significant difference between the two groups (Wilcoxon rank‐sum test, FDR < 0.05; Table S4). 55 gastric microbial pathways were enriched in the *H. pylori*‐positive group, whereas only 2 pathways were more abundant in the *H. pylori*‐negative group, involving in dTDP‐L‐rhamnose biosynthesis and tetrapyrrole biosynthesis (FDR<0.05, Wilcoxon rank‐sum test).

## Discussion

The current study identified *H. pylori* infection‐related changes in gastric microbiome composition and function. The colonization of *H. pylori* in the human stomach significantly decreased the diversity of the gastric microbiome, and the relative abundance of *Stenotrophomonas maltophilia*, *Stenotrophomonas unclassified*, *Variovorax unclassified*, *Chryseobacterium* unclassified, *Comamonas* unclassified, and *Pseudomonas stutzeri* was decreased in the stomach of *H. pylori‐positive* individuals. Furtherly, we also investigated the difference in microbial metabolic pathways between *H. pylori‐positive* and *H. pylori*‐negative groups and found most differential pathways were enriched in the *H. pylori‐positive* group. Additionally, we also explored the association between gastritis and the gastric microbiome and found that the correlation between gastritis and the gastric microbiome is slight.

We identified the main phyla in our gastric swab samples, including *Proteobacteria*, *Bacteroidetes*, *Firmicutes*, and *Actinobacteria*; these phyla together accounted for 99.82% of the total abundance in all samples averagely, which is in line with previously reported results [23]. In gastric biopsy samples from children, the total abundance of *Proteobacteria*, *Bacteroidetes*, *Firmicutes*, and *Actinobacteria* was also greater than 97% in both *H. pylori*‐positive and *H. pylori*‐negative individuals [12], indicating the overall composition of the gastric microbiome is relatively similar in adults and children. In our samples, *Proteobacteria* dominated the gastric microbial community, and its proportion increased in *H. pylori‐infected* individuals, which was mainly caused by the considerable abundance of *H. pylori* in the stomach of infected patients.

At the species level, we found that the colonization of *H. pylori* considerably altered the gastric microbial community structure. The most obvious effect of *H. pylori* infection on the gastric microecological system is the reduction in gastric microbial species diversity; higher Shannon’s diversity can be observed in individuals with lower *H. pylori* abundance compared to those with higher *H. pylori* abundance. The negative correlation between *H. pylori* abundance and other non‐*H. pylori* microbial diversity may be related to physiological changes in the stomach, where *H*. *pylori* gains a survival advantage under acidic conditions in the early stages of chronic *H. pylori* infection and becomes the dominant gastric bacteria leading to a decrease in the biodiversity of the gastric microbiome. However, after the persistent infection of *H. pylori*, which may cause gastric atrophy, an increase in the gastric pH in turn creates a survival environment for other bacteria that are not able to grow in the acidic environment, and eventually inhibits *H. pylori* growth through nutritional competition or other unknown mechanisms [24]; then, gastric biodiversity increases again; and therefore, the microbial composition of the stomach also shows dynamic changes throughout the process of *H. pylori* colonization and is relevant to the pathological changes in the stomach [24, 25]. Microbial interaction network analysis has shown that negative interactions of *H. pylori* with other bacterial species play a dominant role in network changes [26]. The current study suggests that *H. pylori* plays a critical role in the onset stage of gastric cancer, but not in the progression stage. The progression of gastric cancer may be associated with the growth of other non‐*H. pylori* bacteria in the stomach, which need to be further validated [27].

*H. pylori* infection is an important risk factor for gastritis, which is characterized by neutrophilic infiltration, and the role of other non‐*H. pylori* bacterial species in the human stomach during the onset and progression of gastritis remains unclear. We compared the gastric microbiome composition between no gastritis, chronic gastritis, and chronic active gastritis groups and found that non‐*H. pylori* species in the stomach were not associated with gastritis, which indicates the elevated risk of gastric diseases may mainly be contributed by *H. pylori*. Gantuya et al reported that *Streptococcus* species and *Haemophilus parainfluenzae* were associated with the increased risk of *H. pylori*‐negative gastritis in the East Asian population [28]. However, we did not replicate this result. The potential role of non‐*H. pylori* species in the pathogenic process of gastritis still requires further investigation in a larger cohort. Additionally, since *H. pylori* dominate the gastric microbial community after its colonization, the increased DNA mass of *H. pylori* may decrease the DNA mass of non‐*H. pylori* species, which thus biases the between‐species interaction analysis and causes loss of non‐*H. pylori* species diversity; the high depth sequencing and sensitive caption of non‐*H. pylori* microbial species are required in the future study.

## Conclusions

The overall composition of the gastric microbiota in *H. pylori‐infected* individuals was distinctly different from that of the negative controls, *H. pylori* becomes the dominant species after colonizing the stomach, and *H. pylori* infection‐associated microbial species were identified (*P* < 0.05, Wilcoxon rank‐sum test). Furthermore, 55 gastric microbial pathways were enriched in the *H. pylori*‐positive group, whereas only 2 pathways were more abundant in the *H. pylori*‐negative group, involving dTDP‐l‐rhamnose biosynthesis and tetrapyrrole biosynthesis (*P* < 0.05, Wilcoxon rank‐sum test). Gastritis was not associated with non‐*H. pylori* species in the stomach (*P* > 0.05, Wilcoxon rank‐sum test). This study revealed the taxonomic and functional alterations in the gastric microbiome associated with *H. pylori* infection in the Chinese population, which provides an insight into gastric microbial interactions and their potential role in the pathological process of gastric diseases.

## Conflict of interest

The authors declare no conflict of interest with respect to the authorship and/or publication of this article.

## Authors’ contributions

JM and XZ designed the study. JM and XZ managed the project. YW, LD, and XZ contributed to the acquisition of sample and clinical data. DW, TZ, CW, YL, and YT contributed to data generation and performed the data analyses. JL contributed to DNA extraction and sequencing. DW drafted the manuscript. All authors revised the manuscript and approved the final manuscript.

## Supporting information


**Table S1.** Metadata of samples in this study.
**Table S2.** Comparison of species relative abundance between *H. pylori‐positive* and negative groups.
**Table S3.** Comparison of species relative abundance between no gastritis, chronic gastritis, and chronic active gastritis groups.
**Table S4.** Comparison of gastric microbial pathway relative abundance between *H. pylori‐positive* and negative groups.

## Data Availability

The data that support the findings of this study have been deposited into CNGB Sequence Archive (CNSA) [29] of the China National GeneBank DataBase (CNGBdb) [30] with accession number CNP0002239.
